# Aggregation behavior and reproductive compatibility in the family Cimicidae

**DOI:** 10.1038/s41598-017-12735-3

**Published:** 2017-10-13

**Authors:** Zachary DeVries, Russell Mick, Ondřej Balvín, Coby Schal

**Affiliations:** 1 Department of Entomology and Plant Pathology, North Carolina State University, Raleigh, North Carolina USA; 2 W.M. Keck Center for Behavioral Biology, North Carolina State University, Raleigh, North Carolina USA; 3 Department of Ecology, Faculty of Environmental Sciences, Czech University of Life Sciences, Prague, Kamýcká Czech Republic; 4 Center for Human Health and the Environment, North Carolina State University, Raleigh, North Carolina USA

## Abstract

Bed bugs (*Cimex lectularius*) provide a unique opportunity to understand speciation and host-associated divergence in parasites. Recently, two sympatric but genetically distinct lineages of *C. lectularius* were identified: one associated with humans and one associated with bats. We investigated two mechanisms that could maintain genetic differentiation in the field: reproductive compatibility (via mating crosses) and aggregation fidelity (via two-choice sheltering assays). Effects were assessed at the intra-lineage level (within human-associated bed bugs), inter-lineage level (between human- and bat-associated bed bugs), and inter-species level (between *C. lectularius* and *Cimex pipistrelli* [bat bug]). Contrary to previous reports, bed bugs were found to be reproductively compatible at both the intra- and inter-lineage levels, but not at the inter-species level (although three hybrids were produced, one of which developed into an adult). Lineage- and species-specific aggregation fidelity was only detected in 8% (4 out of 48) of the aggregation fidelity assays run. These results indicate that under laboratory conditions, host-associated lineages of bed bugs are reproductively compatible, and aggregation pheromones are not capable of preventing gene flow between lineages.

## Introduction

The common bed bug, *Cimex lectularius* L., offers a unique system for studies of incipient speciation for several reasons. First, bed bug populations undergo dramatic expansion and contraction cycles, as infestations often start from very small propagules^1–3^, rapidly expand within and between apartments with extensive inbreeding, and then undergo repeated population bottlenecks because of human interventions and founder effects^4^. Second, as obligate ectoparasites they rely on their host for nutrition^5^, and therefore must adapt to any changes in host behavior and physiology, or alternatively, adapt to a new host. Bed bugs are polyphagous, accepting a broad range of hosts from birds to humans^5^, and persistent association with a single host can result in adaptive evolution that makes alternate hosts less acceptable^6,7^. Finally, bed bugs have an ancestral association with bats, predating their more recent association with humans^8^. Balvin *et al*.^8^ recently studied a central European bat-associated (BA) lineage of *C. lectularius* that overlaps geographically with a human-associated (HA) lineage. Despite being found in the same environment, there is little to no apparent gene flow between the two lineages across Europe^8–10^. These factors – small propagule size, extensive inbreeding, recurrent bottlenecks and founder effects, pressure to adapt to changes in host culture, behavior and physiology, and the existence of differentiated host-associated lineages in sympatry – produce an optimal scenario for sympatric speciation^4^.

The apparent lack of gene flow between the HA and BA *C. lectularius* lineages suggests that complete or incomplete reproductive isolation might have evolved. Indeed, Wawrocka *et al*.^11^ showed that interbred HA and BA bed bugs were incapable of producing hybrids, suggesting complete reproductive isolation, no gene flow, and that *C. lectularius* has differentiated into two species. Our preliminary results significantly departed from these findings, however, suggesting differences related to methodology or physiology of the bed bugs.

Incomplete reproductive isolation, with lineage differentiation occurring in allopatry or in sympatry, is expected to result in hybrids with lower fitness, and reinforcement is expected to increase reproductive isolation and reduce inter-lineage hybrids^12^. Mechanisms that minimize gene flow between differentiated lineages may be physiological and/or behavioral, and prezygotic and/or post-zygotic. Determining what factor(s) are responsible for maintaining genetic differences among lineages can be difficult, as many factors can be at play and environmental conditions can modulate their effects.

Bed bugs tend to aggregate, and their fidelity to lineage-specific or even population-specific aggregations might reduce gene flow and cause lineages to differentiate. Aggregations serve a variety of functions in insects, and in bed bugs aggregations have been shown to reduce water loss^13^ and accelerate development^14^. Other benefits accrued in aggregations, as shown in the German cockroach, might be nutrient sharing, for example through coprophagy^15^, or faster reproduction^16^. Aggregation sites also provide potential arenas for mate location^5^. Since the formation and maintenance of aggregations are guided by aggregation pheromones^17^, it is reasonable to expect that aggregation cues should diverge in species and lineages that produce inviable or less fit hybrid offspring. Balvín *et al*.^18^ tested this hypothesis and did not find evidence of aggregation fidelity. However, in these assays only adult males were used to bioassay aggregation preferences, thus it is necessary to assay both females as shelter conditioners and aggregation responders. Additionally, only inter-lineage preference (HA vs. BA) were evaluated. The extent species-level aggregation fidelity also need to be considered given the co-habitation of BA *C. lectularius* and *Cimex pipistrelli* in bat roosts (O.B. field collection, 2014).

To evaluate the role of lineage- and species-specific aggregation pheromones in guiding fidelity to aggregation sites and possibly in maintaining genetic differentiation of bed bug lineages, bed bugs were tested to determine if they preferred aggregating with their own or other populations. Additionally, reproductive compatibility was assessed among lineages tested for aggregation fidelity to determine if these lineages were reproductively isolated. The results are interpreted in relation to the evolution of HA and BA bed bug lineages and the factors that facilitate the maintenance of these lineages.

## Materials and Methods

### Experimental Animals

Three populations of *C. lectularius* with two different host associations were used: Winston Salem (WS-HA) and Jersey City (JC-HA) were both collected in close association with humans (hence, HA, human associated). The WS-HA population was collected in 2008 from beds and couches in an apartment in Winston Salem, North Carolina, USA. The JC-HA population was collected in 2008 from an apartment in Jersey City, New Jersey, USA. The third population, Moravičany (MO-BA, bat associated), was collected in 2014 living in close association with a bat colony (*Myotis myotis*) in Moravicany, Czech Republic. In addition, a population of bat bugs, *Cimex pipistrelli* (Cp-BA) collected in Dubá, Czech Republic in 2014, was also used. Together, these populations allowed for intra- (HA vs. HA) and inter-lineage (HA vs. BA) comparisons, as well as inter-species comparisons (*C. lectularius* vs. *C. pipistrelli*).

All bed bugs and bat bugs were reared in the laboratory in 168 cm^2^ plastic cylinders with cardstock paper substrate at 27 °C and ~50% RH on a 12:12 light:dark cycle. Bed bugs and bat bugs were fed defibrinated rabbit blood in an artificial feeding system. This system relied on a heated water bath (B. Braun Biotech Inc., Allentown, PA) to circulate water heated to 37 °C through a custom-made water jacketed glass feeder, with bed bugs feeding through an artificial membrane (Nescofilm, Karlan, Cottonwood, AZ, USA). It is important to note that under these conditions all bed bugs and bat bugs experienced the same environmental conditions and blood meal type.

### Reproductive Compatibility

Reproductive compatibility was assessed by pairing virgin adults from different populations and looking for offspring. Fifth instars of each population were isolated and allowed to eclose, ensuring newly emerged adults remained unmated. Bed bugs were recombined by sex and population, then fed. One week after feeding, bed bugs were fed again, then individual virgin males and females were combined in 7.5 ml glass vials with a paper shelter and allowed 6 d to lay eggs. Preliminary trials revealed six days as enough time to reflect reproductive output, and no differences were found when comparing results at 6d or 10 d. Only adults that fed to repletion (fully engorged) were used for bioassays. Adults were then removed and vials were monitored for the number of nymphs that hatched after 2 weeks. Sample sizes were 16–18 pairs for intra-lineage assays (WS-HA vs. JC-HA), 9–10 pairs for inter-lineage assays (WS-HA vs. MO-BA), and 15 pairs for inter-species assays (WS-HA vs. Cp-BA). All assays were female-centric, with comparisons made between individual females of the same population/species mated to males of either the same or a different population/species. In addition, all assays within each level of testing (intra-lineage, inter-lineage, inter-species) were run in parallel.

### Aggregation Preference Assays

Aggregation assays were conducted in plastic petri dishes (d = 60 mm; Corning Life Sciences, Durham, NC, USA), with the bottom etched to facilitate bed bug movement and lids on to prevent escape. Two white cardstock paper tents (30 mm × 15 mm, 110 lb weight, Georgia Pacific LLC, Atlanta, GA) were placed adjacent to each other in a symmetrical design in each arena. Each paper tent was conditioned by one fed bug of one of the following groups: male WS-HA, female WS-HA, male JC-HA, female JC-HA, male MO-BA, female MO-BA, male Cp-BA, and female Cp-BA. Bed bugs were fed in large cohorts (50–100 bugs) divided by sex and population, then moved to conditioning vials following feeding. Conditioning occurred in 20 ml glass vials, where the paper tent served as the only shelter for the bugs. Vials had mesh tops to allow for air flow and ventilation. Assay development and validation was run with one population (WS-HA) to establish a conditioning protocol that was sufficient to elicit aggregation. In these assays a conditioned tent was tested against a clean, unconditioned tent (treated identically in all ways to the conditioned tents). The number of bugs and conditioning time were varied to ensure the experimental conditions were sufficient to assess aggregation.

In validated assays, each tent was conditioned for one day, with a new tent conditioned every day for four days following feeding (each bed bug used for conditioning produced four tents over four days after feeding). Two-choice aggregation preference assays were run independently for male and female responders for the following combinations of conditioned tents: WS-HA male vs. JC-HA male, WS-HA female vs. JC-HA female, WS-HA male vs. MO-BA male, WS-HA female vs. MO-BA female, WS-HA male vs. Cp-BA male, and WS-HA female vs. Cp-BA female. After the arena was set up, a single adult of one of the two populations being tested was introduced into the center of the arena. Male and female bed bugs were assayed separately. Bioassayed adults were fed 2 d prior to the start of each experiment to minimize their propensity to seek a host and maximize their tendency to seek a suitable shelter. All assays were set up 4 h after lights-off and responders were then allowed the remaining 8 h of the scotophase to move freely in the dark around the arena and sample both tents. The final position of each bed bug was recorded 3–4 h after lights-on to maximize the number of bugs fully arrested, with non-responders defined as those not located on or under either tent. Assays were always run in blocks, with identical numbers of bed bugs of each population tested for each choice combination.

Male aggregation preference was also assessed on tents conditioned by groups of bed bugs or bat bugs, to see if results differed with increased levels of conditioning. The same protocol used for single-bug conditioned tents was used, with the only difference being that each tent was conditioned by 10 adult male or female bed bugs for 5 d following feeding, allowing us to test substrates that were more heavily conditioned. In addition, and in response to observations from the group-conditioning assays, a final aggregation preference assay was run comparing aggregation preference of JC-HA and WS-HA males for tents unequally conditioned by ten WS-HA or five JC-HA females for five days. This assays was intended to simulate potential field conditions, where co-habitating populations/species likely have different population sizes. Thus, these assays evaluated quantitative versus qualitative preferences.

### Data Analysis

Student’s *t*-test was used to compare the number of emergent nymphs between hetero- and homogeneous crosses involving the same female. Aggregation preferences were tested by Chi Square analysis, with the null hypothesis of no aggregation-site preference, or a 1:1 shelter tent choice. All analysis was performed in SAS 9.4^19^ (SAS Institute, Cary, NC, USA).

### Data Availability

All data and statistical code used in the current study are available from the corresponding author upon reasonable request.

## Results

### Reproductive Compatibility

**Figure 1:**
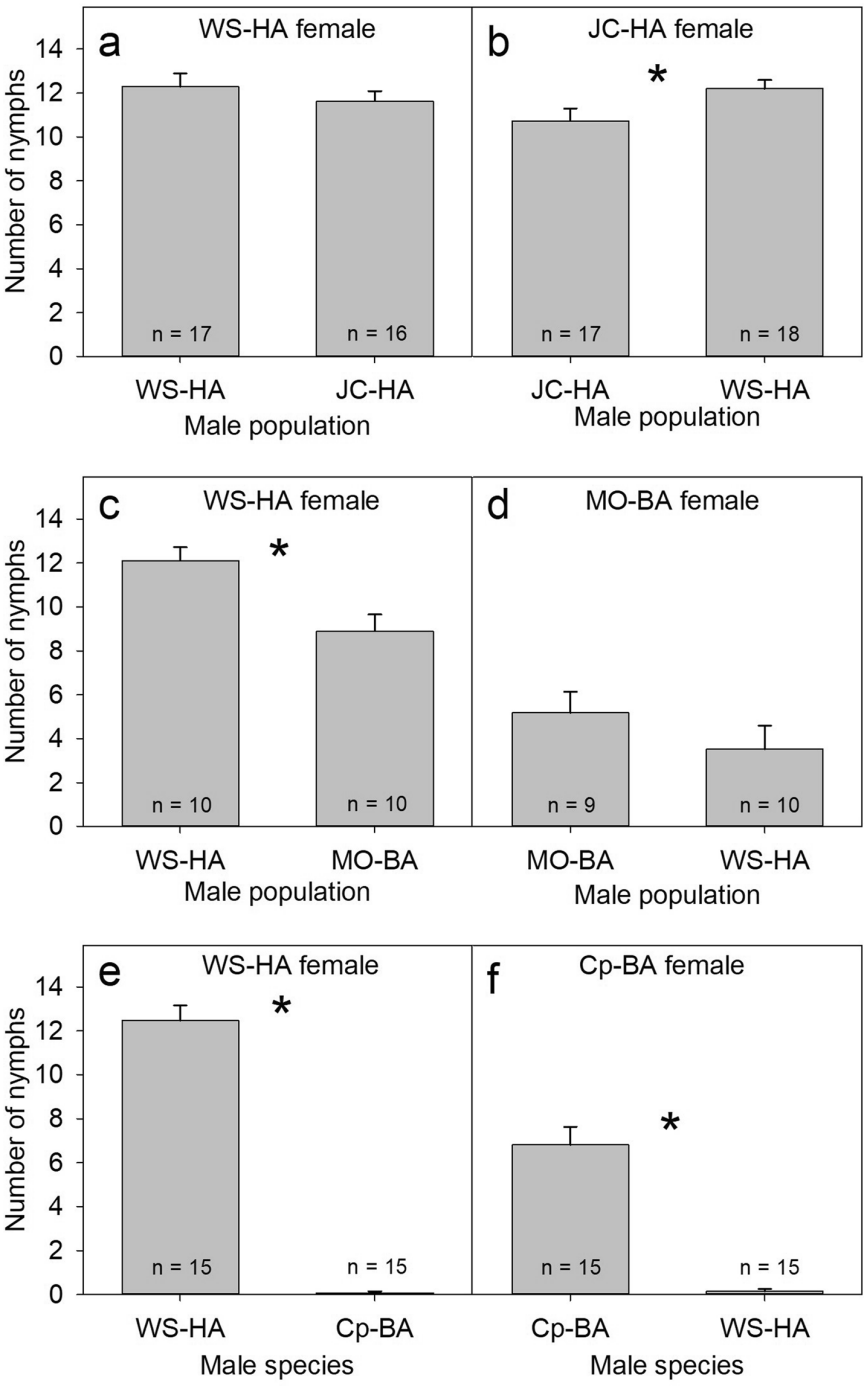
Reproductive compatibility as indicated by the number of nymphs produced between homogeneous and heterogeneous crosses. Comparisons are focused on each female, and are made within a lineage (WS-HA vs. JC-HA; (**a**,**b**)), between lineages (WS-HA vs. MO-BA; (**c**,**d**)), and between species (WS-HA vs. Cp-BA; (**e**,**f**)). Significance is based on Student’s *t*-test (*p* < 0.05), and indicated by an asterisk (*).

The population from which males originated had no effect on the number of viable offspring produced by WS-HA females (*t*_31_ = 0.87, *p* = 0.3912, Fig. 1a). However, JC-HA females produced more offspring when mated to WS-HA males than with their own JC-HA males (*t*_33_ = 2.14, *p* = 0.0397, Fig. 1b).

Winston Salem (HA) females produced significantly more offspring when mated to their own WS-HA males than to MO-BA males (*t*_18_ = 3.28, *p* = 0.0042, Fig. 1c). However, the number of offspring produced by MO-BA females was unaffected by the male’s lineage (*t*_17_ = 1.16, *p* = 0.2627, Fig. 1d).

Bed bugs (*C. lectularius*, WS-HA population) and bat bugs (*C. pipistrelli*, Cp-BA) were not reproductively compatible (*t*_28_ = 17.91, *p* < 0.0001, Fig. 1e; *t*_18_ = 8.04, *p* < 0.0001, Fig. 1f). Only three *C. lectularius*-*C. pipistrelli* hybrid nymphs were observed, one of which was dead during the initial observation. Of the remaining two nymphs, both were offered blood, but only one survived to maturity – an adult male that originated from a female *C. pipistrelli* mated to a male *C. lectularius*. However, the surviving hybrid failed to sire any offspring when maintained separately with unmated females of either parental species. Notably, the bat-associated females (MO-BA, Cp-BA) were significantly less fecund on rabbit blood than the human-associated *C. lectularius* even in conspecific pairings.

### Conditioned vs. Control Shelters

**Figure 2:**
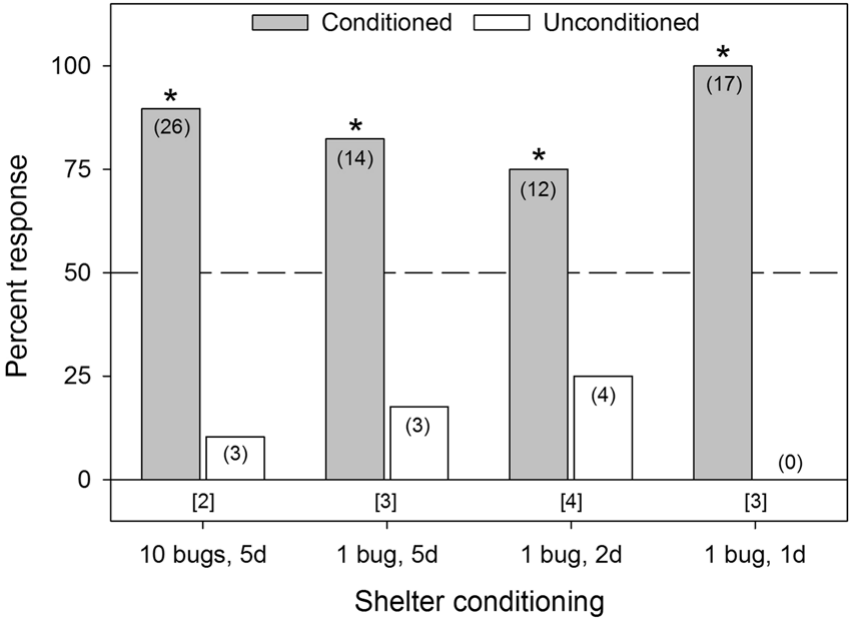
Aggregation responses of individual male bed bugs (*C. lectularius*) of the human-associated WS strain to tent shelters either conditioned by various numbers of WS males for various amounts of time or unconditioned (clean controls). The number of individual bugs that responded to each choice is indicated in parenthesis. The number of non-responders is indicated in brackets below each set of choices for each assay. An asterisk (*) indicates significant choice of one shelter over the other, based on the Chi-square test (*p* < 0.05).

To confirm that bed bugs could detect and preferentially arrest under bed bug-conditioned paper shelters, experiments were run comparing WS-HA male-conditioned shelters vs. unconditioned shelters. Bed bugs displayed a strong preference for conditioned shelters, when conditioned by 10 bed bugs for five days (χ^2^_1,29_ = 18.24, *p* < 0.0001), or one bed bug for either five days (χ^2^_1,17_ = 7.12, *p* = 0.0076), two days (χ^2^_1,16_ = 4.00, *p* = 0.0455), or one day (χ^2^_1,17_ = 17.00, *p* < 0.0001; Fig. 2). Based on these results, further assays were conducted with shelters conditioned by one bug for one day and 10 bugs for five days.

### Intra-lineage Aggregation Preferences

**Figure 3:**
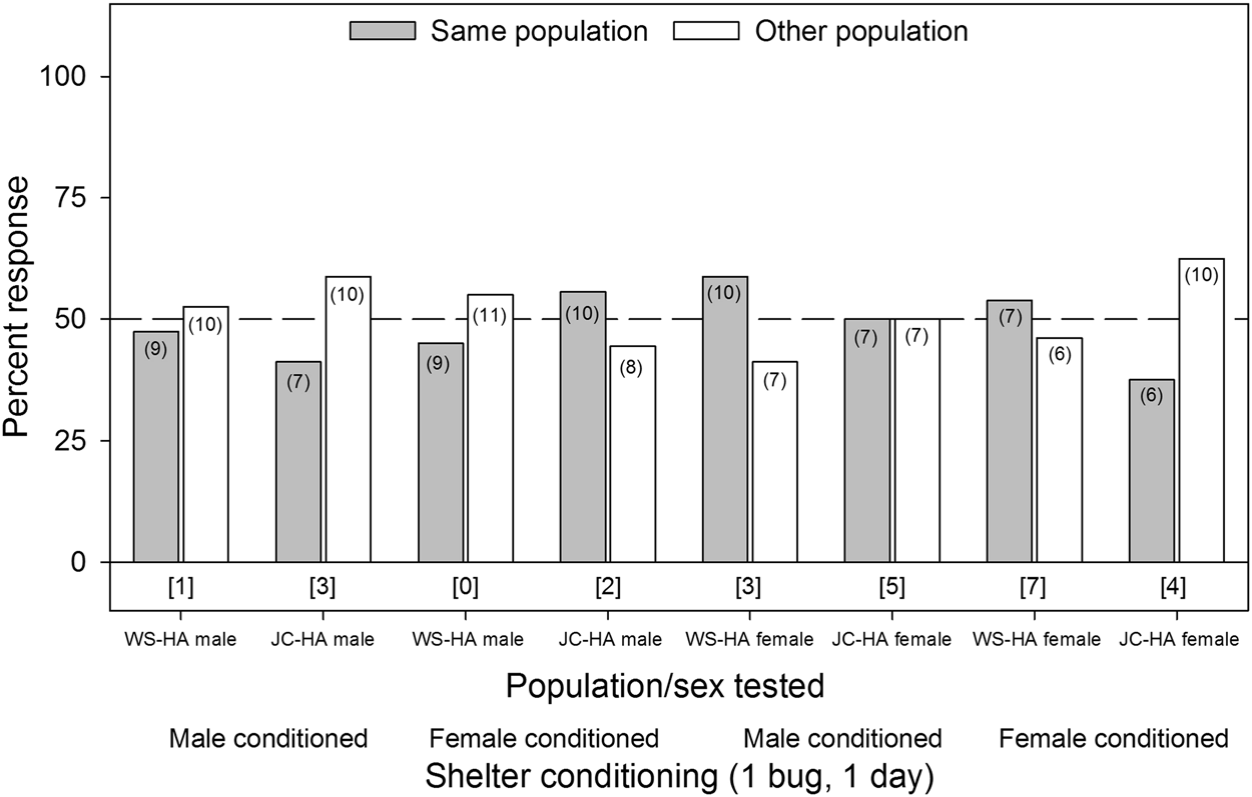
Aggregation responses of individual bed bugs (*C. lectularius*) of the human-associated WS and JC strains to shelters conditioned for 24 hrs by single members of their own population or the other population. The population/sex tested and the shelter conditioning information (male or female conditioned) are listed below the figure. The number of individual bugs that responded to each choice is indicated in parenthesis. The number of non-responders is indicated in brackets below each set of choices for each assay. All the assays did not show significant differences in the choice of one shelter over the other, based on the Chi-square test (*p* ≥ 0.3173 for all assays).

**Figure 4:**
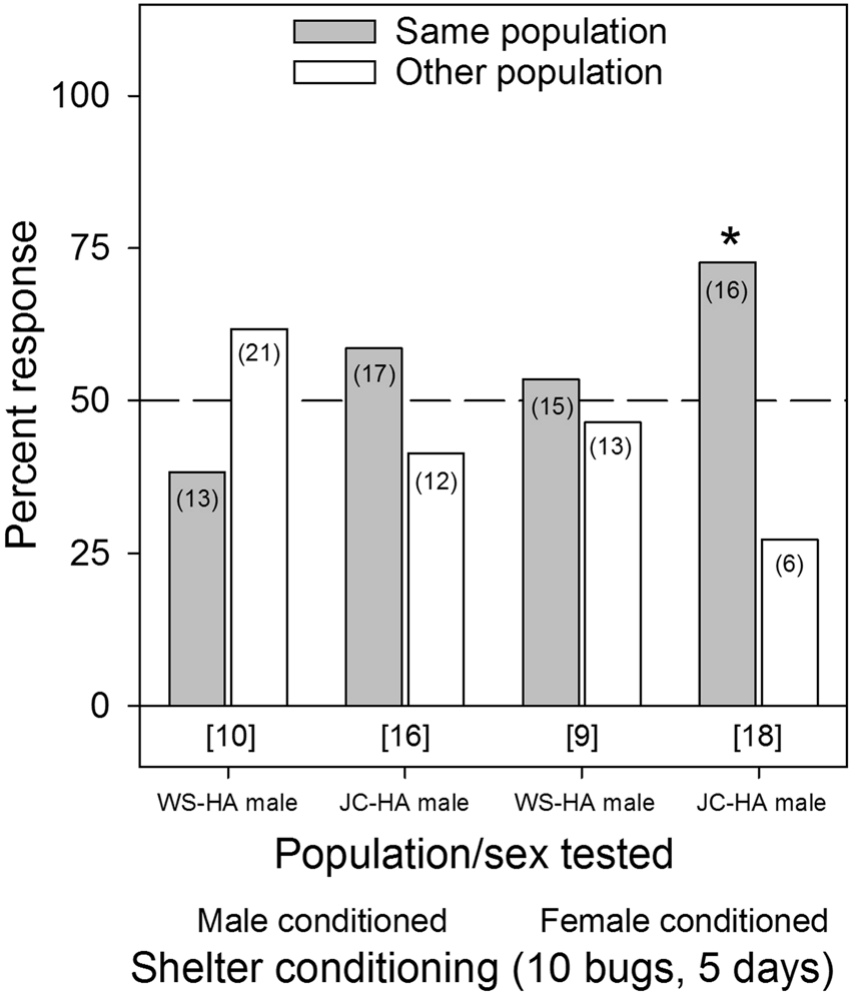
Aggregation responses of individual male bed bugs (*C. lectularius*) of the human-associated WS and JC strains to shelters conditioned for 5 days by 10 adult male or female bed bugs of their own population or the other population. The population/sex tested and the shelter conditioning information (male or female conditioned) are listed below the figure. The number of individual bugs that responded to each choice is indicated in parenthesis. The number of non-responders is indicated in brackets below each set of choices for each assay. An asterisk (*) indicates significant choice of one shelter over the other, based on the Chi-square test (*p* < 0.05).

Intra-lineage aggregation preference was not detected in any of the single bed bug conditioning assays, regardless of the sex of the responder or the sex of the bugs used to condition each tent (*p* ≥ 0.3173 for all assays, Fig. 3). Assays were also conducted that involved allowing 10 adult males or females to condition each tent for 5 d. These assays indicated some aggregation preference, specifically for the JC-HA males when tested on female conditioned shelters (χ^2^_1,13_ = 4.55, *p* = 0.0330, Fig. 4). However, no other groups tested showed significant aggregation site preference (*p* ≥ 0.1701, Fig. 4).

### Inter-lineage Aggregation Preferences

**Figure 5:**
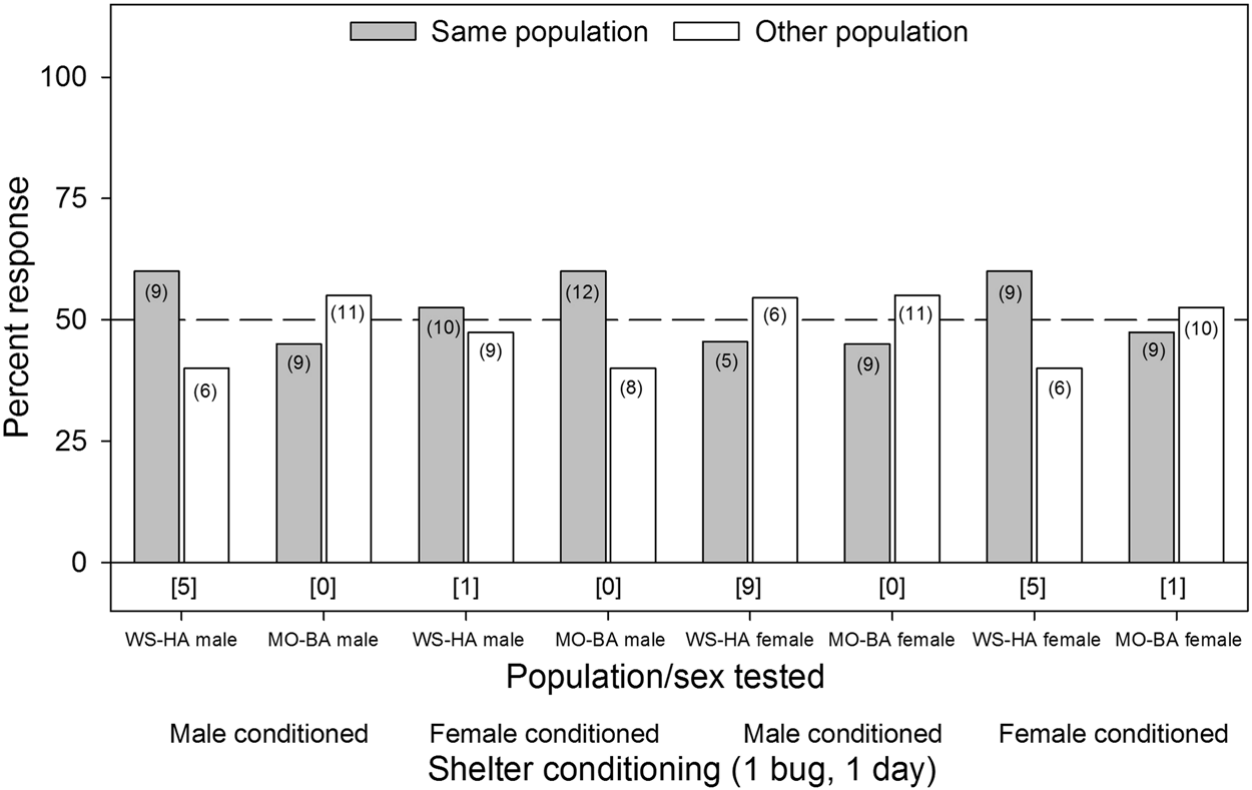
Aggregation responses of individual bed bugs (*C. lectularius*) of the human-associated WS strain and the bat-associated MO strains to shelters conditioned for 24 hrs by single members of their own lineage or the other lineage. The population/sex tested and the shelter conditioning information (male or female conditioned) are listed below the figure. The number of individual bugs that responded to each choice is indicated in parenthesis. The number of non-responders is indicated in brackets below each set of choices for each assay. All the assays did not show significant differences in the choice of one shelter over the other, based on the Chi-square test (*p* ≥ 0.3711 for all assays).

**Figure 6:**
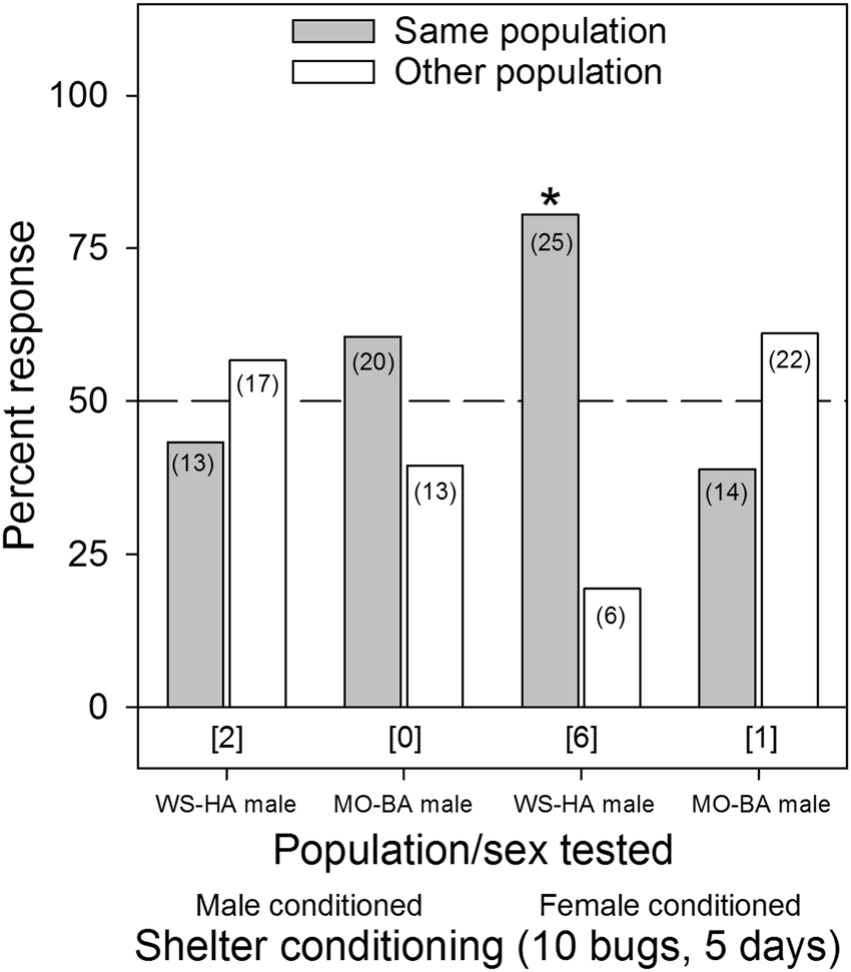
Aggregation responses of individual bed bugs (*C. lectularius*) of the human-associated WS strain and the bat-associated MO strain to shelters conditioned for 5 days by 10 adult male or female bed bugs of their own lineage or the other lineage. The population/sex tested and the shelter conditioning information (male or female conditioned) are listed below the figure. The number of individual bugs that responded to each choice is indicated in parenthesis. The number of non-responders is indicated in brackets below each set of choices for each assay. An asterisk (*) indicates significant choice of one shelter over the other, based on the Chi-square test (*p* < 0.05).

No evidence for lineage preference was detected in any of the experiments with a single bed bug-conditioned tent, regardless of the sex of the responder or the sex of the bugs used to condition each tent (*p* ≥ 0.3711, Fig. 5). For the assays involving tents conditioned by 10 bed bugs for five days, there was a strong preference by WS-HA males for female-conditioned shelters of their own WS-HA population over the MO-BA population (χ^2^_1,13_ = 11.65, *p* = 0.0006, Fig. 6). But no other groups tested showed significant aggregation site preferences (*p* ≥ 0.1824, Fig. 6).

### Inter-Species Aggregation Preferences

**Figure 7:**
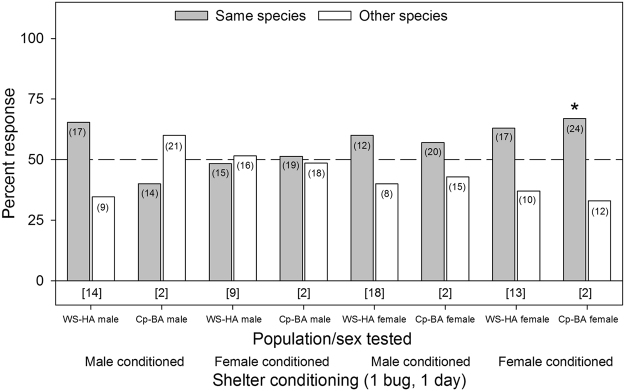
Aggregation responses of individual bed bugs (*C. lectularius*, WS-HA strain) and bat bugs (*C. pipistrelli*, Cp-BA) to shelters conditioned for 24 hrs by a single member of their own species or the other species. The population/sex tested and the shelter conditioning information (male or female conditioned) are listed below the figure. The number of individual bugs that responded to each choice is indicated in parenthesis. The number of non-responders is indicated in brackets below each set of choices for each assay. An asterisk (*) indicates significant choice of one shelter over the other, based on the Chi-square test (*p* < 0.05).

**Figure 8:**
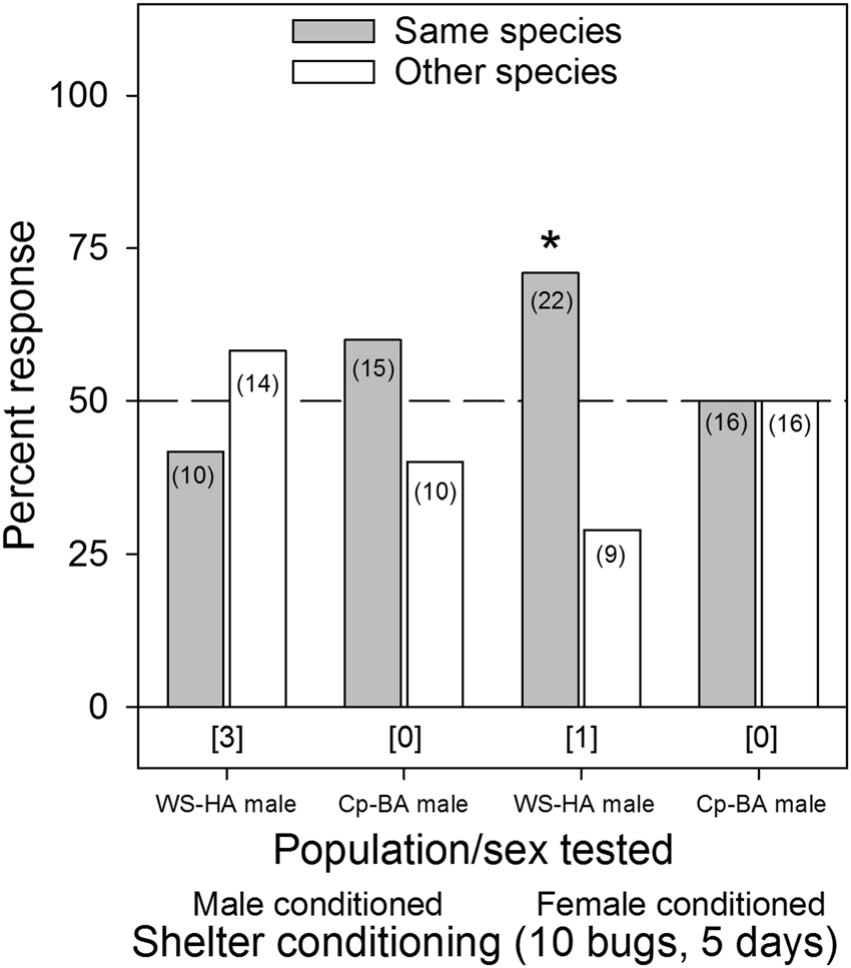
Aggregation responses of individual bed bugs (*C. lectularius*, WS-HA strain) and bat bugs (*C. pipistrelli*, Cp-BA) to shelters conditioned for 5 days by 10 adult male or female bed bugs of their own species or the other species. The population/sex tested and the shelter conditioning information (male or female conditioned) are listed below the figure. The number of individual bugs that responded to each choice is indicated in parenthesis. The number of non-responders is indicated in brackets below each set of choices for each assay. An asterisk (*) indicates significant choice of one shelter over the other, based on the Chi-square test (*p* < 0.05).

Only one of the eight single bed bug conditioning assays (Cp-BA female selecting female conditioned tents) showed significant species-specific aggregation preference (χ^2^_1,36_ = 4.00, *p* = 0.0455), with all other assays being non-significant (*p* ≥ 0.1167, Fig. 7). For the assays involving tents conditioned by 10 bugs for five days, only the *C. lectularius* WS-HA males showed significant preference for female-conditioned shelters of their own species over the *C. pipistrelli* female conditioned tents (χ^2^_1,31_ = 5.45, *p* = 0.0196), with all other assays showing no aggregation preferences (*p* ≥ 0.3173, Fig. 8).

### Effects of Differential Conditioning on Aggregation Preferences

**Figure 9:**
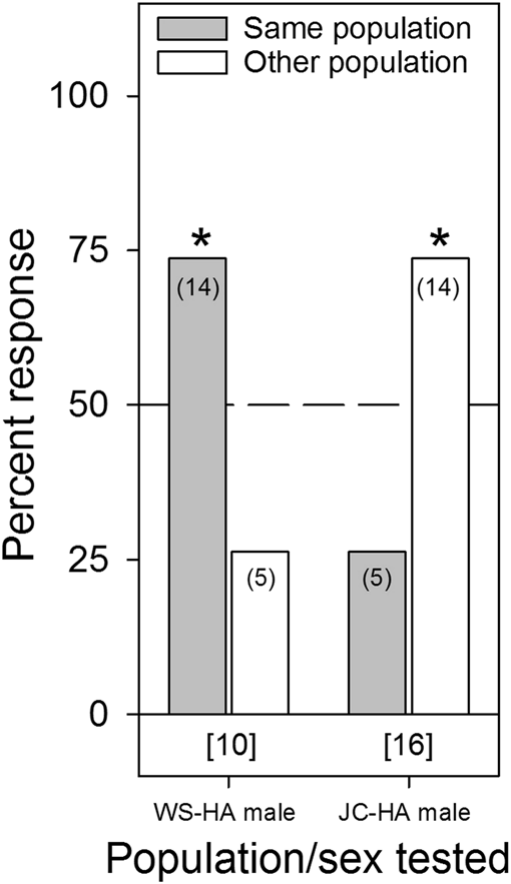
Aggregation responses of individual bed bugs (*C. lectularius*) of the human-associated WS and JC strains to shelters unequally conditioned for 5 days by either 10 WS males or 5 JC males. The population/sex tested is listed below the figure. The number of individual bugs that responded to each choice is indicated in parenthesis. The number of non-responders is indicated in brackets below each set of choices for each assay. An asterisk (*) indicates significant choice of one shelter over the other, based on the Chi-square test (*p* < 0.05).

We suspected that the inconsistent, unidirectional aggregation preferences observed when shelters were conditioned by 10 bed bugs were due to quantitative rather than qualitative differences in aggregation pheromones on conditioned tents. Therefore, we conducted an additional bioassay to assess the effects of differentially conditioned shelters on aggregation preferences. Males of WS-HA and JC-HA both preferentially selected tents conditioned by 10 WS females over tents conditioned by five JC females (χ^2^_1,31_ = 4.26, *p* = 0.0389, Fig. 9).

## Discussion

Human- and bat-associated lineages of *C*. *lectularius* were reproductively compatible in our experiments. Although there may be some costs to inter-lineage mating, such as reduced fecundity, the results of our study indicate that gene flow is possible between these two host-associated lineages. These results deviate radically from a previous study that showed no egg production for any crosses between HA and BA bed bugs^11^. These disparate findings could be attributed to several methodological differences between the two studies. A major difference was that Wawrocka *et al*.^11^ used bugs that were collected in the field as nymphs and tested as same generation adults, whereas bugs in our experiments were acclimated to the laboratory for over a year. Two important changes could have occurred during the >1 year in the laboratory: adaptation to rabbit blood and homogenization of microbiomes between strains. In Wawrocka *et al*.^11^ a diet switch occurred in the 3^rd^ instar or later, whereas in our experiments all the bed bugs had acclimated to rabbit blood over at least 8 generations (estimated based on a development time of 31 d at 27 °C^5^, and at least one year in culture prior to testing, with some adjustment for slower development at colony inception). Switching diets during development (even among diets that are readily accepted by a species) has been shown to negatively affect development in phytophagous generalists^20,21^. Human-associated bed bugs have also been shown to develop poorly when switching diets from human blood to bat blood^9^, although it is unclear how the timing of this switch (early or late in development) affects growth and reproduction. A common food (rabbit blood) fed to both lineages in our experiments could have also homogenized their gut microbiomes more so than in Wawrocka *et al*.’s experiments^9^. *Cimex* species harbor *Wolbachia* as an obligatory symbiont, and *Wolbachia* and other endosymbionts have been shown to play significant roles in cytoplasmic incompatibility and reinforcement of speciation^22–25^. It is possible that long-term colonization and feeding on a common food source homogenized the microbial communities and eliminated reproductive incompatibility between the bed bug lineages. Nevertheless, it is important to note that even after such long-term adaptation to rabbit blood, both the BA lineage (represented by MO-BA) and *C. pipistrelli* (Cp-BA) had lower fecundity, even in homogeneous crosses (Fig. 1). The reasons behind the low fecundity observed in BA bed and bat bugs is unknown, but we speculate it is either due to physiological differences or laboratory conditions.

Previous work has shown that *C. lectularius* and *C. hemipterus* can mate and produce hybrids in the laboratory, although hybrids generally fail to mature^26^. To the best of our knowledge, our results represent the first documented successful hybridization between *C. lectularius* and *C. pipistrelli*. A single hybrid was reared to an adult male, but was unable to sire offspring with females of either of the two parental species. It is important to investigate this phenomenon with greater sample sizes to determine whether some fertile hybrids might result from this cross. Viable inter-species hybrids could constitute an epidemiological bridge between bats and humans, and significantly affect the public health importance and pest status of both species.

Population genetics studies indicate that the human- and bat-associated lineages are genetically distinct with no apparent gene flow between them^8,10^. Although they may be considered sympatric within human-built structures, it is likely that HA and BA bugs are separated by ecological and behavioral barriers. Ecological separation may be imposed by the divergent niches of the two respective hosts – chimneys and attics for BA bugs and residential rooms, especially bed rooms, for HA bugs. Behavioral barriers may include differential responses to host odors and pheromones, including aggregation pheromones. We hypothesized that the two lineages would exhibit lineage-fidelity in their aggregation responses. To our surprise however, aggregation fidelity was not observed in any of the populations, lineages, or even species tested. These results suggest that there are no preferences for lineage-specific aggregation pheromones, and that aggregation preferences do not represent a substantial behavioral isolating mechanism.

Our results are consistent with the results of Balvín *et al*.^18^, who documented similar behavioral responses for adult males, and we further extended these findings to females and to lack of preferences between *C. lectularius* and *C. pipistrelli*. The results from the interspecific aggregation assays (*C. lectularius* vs. *C. pipistrelli*) provide strong evidence that in the absence of species-level aggregation fidelity, it is unlikely that lineage-level aggregation preferences alone would be sufficient to maintain genetic isolation of the two lineages. Similar to the reproductive compatibility assays, the results of the aggregation assays might have been affected by the fact that all bugs were reared on the same diet in the same manner and thus likely shared environmental microbes and possibly even endosymbiotic bacteria. Gut microbes have been shown to influence aggregation in cockroaches^27^ and firebrats^28^. Such effects likely extend beyond these two insects, and possibly to bed bugs. The homogenization of microbiota within the lab could mask any effects that would have otherwise been observed in the field. The effects of environmental factors, and particularly microbes and diet, should be further evaluated for their roles in bed bug aggregation and host-associated differentiation. It is worth noting that in all experiments involving HA and BA bed bugs, HA bed bugs were found to have higher numbers of non-responders that BA bed or bat bugs. This phenomenon could be indicative of behavioral differences among populations or differential responses to conditioned shelters, although at this time we can only speculate on this observation.

Several aggregation assays deviated considerably from the overall pattern of no population- or lineage-specific aggregation fidelity. For example, JC-HA males preferred shelters that were heavily conditioned (10 bugs for 5 days) by their own JC-HA females over WS-HA females (Fig. 4), but this preference was not evident on lightly conditioned (1 bug for 1 day) shelters (Fig. 3). Likewise, WS-HA males preferred their own females’ heavily conditioned shelters over shelters conditioned by either MO-BA females (Fig. 6) or Cp-BA females (but not males) (Fig. 8), but not on lightly conditioned shelters (Figs. 5,7). We suspected that these results were caused by quantitative differences in shelter conditioning (amount of aggregation pheromones deposited onto the conditioned tents) rather than qualitative differences in pheromone preferences among the populations. We tested this hypothesis by titrating the quantitative effects of the aggregation pheromones and comparing aggregation responses to shelters conditioned by 10 or 5 bugs. The results showed that heavier conditioning of the shelters outweighed qualitative differences in the composition of the aggregation pheromones from different HA populations. These results clearly show that bed bugs are more responsive to quantitative differences rather than qualitative differences. Furthermore, these experiments provide additional evidence against the ability of aggregation pheromones to prevent gene flow among lineages or species.

Overall, these results provide strong evidence that under laboratory conditions, HA and BA bed bug lineages are reproductively compatible, and aggregation preferences do not facilitate behavioral reproductive isolation. This suggests that other factors are responsible for the observed genetic divergence among HA and BA bed bug populations in the field. It is still unclear how environmental factors (diet, microbes) influence reproductive compatibility and aggregation fidelity and whether ecological barriers prevent contact between divergent host-associated bed bugs. Therefore, future studies should investigate reinforcement mechanisms that sustain genetic differentiation between these two host-associated lineages of *C. lectularius*.
